# Dual mechanism of Let-7i in tumor progression

**DOI:** 10.3389/fonc.2023.1253191

**Published:** 2023-09-27

**Authors:** Jiapei Zhou, Hongjie Xiang, Zhiqun Cao

**Affiliations:** ^1^ First Clinical Medical College, Shandong University of Traditional Chinese Medicine, Jinan, China; ^2^ Department of Traditional Chinese Medicine, The First Affiliated Hospital of Shandong First Medical University & Shandong Provincial Qianfoshan Hospital, Jinan, China

**Keywords:** Let-7i, cancer, promoter, inhibitor, dual mechanism

## Abstract

Let-7i regulates tumors primarily by binding to the 3′ untranslated region (3′ UTR) of mRNA, which indirectly regulates post-transcriptional gene expression. Let-7i also has an epigenetic function via modulating DNA methylation to directly regulate gene expression. Let-7i performs a dual role by inducing both the promotion and inhibition of various malignancies, depending on its target. The mechanism of Let-7i action involves cancer cell proliferation, migration, invasion, apoptosis, epithelial-mesenchymal transition, EV transmission, angiogenesis, autophagy, and drug resistance sensitization. Let-7i is closely related to cancer, and hence, is a potential biomarker for the diagnosis and prognosis of various cancers. Therapeutically, it can be used to promote an anti-cancer immune response by modifying exosomes, thus exerting a tumor-suppressive effect.

## 1 Introduction

MicroRNA (miRNA) refers to short non-coding RNA with a length of 19–25 nucleotides that functions as a conservative post-transcriptional regulator of gene expression. It recruits Argonaute proteins to form the RNA-induced silencing complex (RISC), which regulates RNA via base-complementary pairing. The combination of miRNA and RISC can inhibit mRNA translation without destroying the stability of mRNA as well as silence unwanted genetic material and transcripts (1, 2). When the miRNA and mRNA involved are entirely complementary, the complex can also mediate mRNA degradation to inhibit transcription (3), thereby regulating the production of the resulting protein. In addition, it has been found that mature miRNAs have the ability to enter the nucleus, directly combine with the original components of gene promoter regions, and contribute to the regulation of non-classical gene transcription (4). MiRNA is involved in almost all biological processes, including cell growth, proliferation, differentiation, metabolism, and the development of organisms (5). Each miRNA binds to hundreds of different mRNAs, and miRNA controls more than half of human protein-coding genes (6). Therefore, the dysregulation of miRNA expression is closely related to the occurrence of various diseases, including cancer (7).

MiRNA biogenesis requires a series of sequential processing events. First, miRNA is transcribed as long primary transcripts (pri-miRNA). This pri-miRNA is subsequently trimmed to 70-nucleotide (nt) pre-miRNAs in the nucleus. Then, the trimmed pre-miRNA is exported to the cytoplasm and synergized by Dicer and Drosha, which are both members of the RNase III superfamily of bidentate nucleases. This cleavage event yields mature miRNA molecules that are approximately 22 nt in length (8–11).

Let-7 was first found in the nematode and identified as a key developmental regulator (12). It is one of the two first known microRNAs (the other one being Lin-4) and the first known human miRNA. The Let-7 family is often present in multiple copies in a genome (13). To distinguish between its multiple subtypes, a letter is placed after Let-7 to represent its various sequences, while numbers at the end of the name indicate that the same sequence exists in multiple genomic locations (13, 14). There are 10 mature Let-7 family sequences in humans that arise from 13 precursor sequences and function in similar ways (13).

Let-7 expression is reportedly downregulated in several human cancers, including esophageal, lung, and breast cancers. As a tumor suppressor, Let-7 miRNA targets various oncogenic molecules (including RAS, HMGA 2, and cell cycle and apoptosis regulators) and exerts its anti-tumor effect by preventing proliferation, promoting apoptosis, inhibiting angiogenesis, and reducing immune surveillance (15–17).

Small differences in the sequence of Let-7 can alter the affinity for its target sequences, thereby resulting in differences in its function or employed mechanism (18). The expression of different family members also varies significantly between tumors. Most Let-7i family members exert an anti-tumor effect to function as tumor suppressors (19), but interestingly, recent studies have found that Let-7i may also act as an oncogene to promote the occurrence and development of cancer (20, 21). Let-7i has been shown to have tumor-suppressive as well as tumor-promoting properties simultaneously. To clarify the specific mechanisms differentiating between the tumor-suppressing and tumor-promoting roles of Let-7i, the current Review summarizes previous studies to provide guidance for further targeted precision therapies in a clinical setting.

## 2 Tumor suppressor function

Let-7i has been widely recognized and studied as a tumor suppressor. Its mechanism of inhibiting tumor development involves not only the modulation of cell proliferation, metastasis, and changes in the tumor cells themselves (such as autophagy, apoptosis, and stem cell properties) but also changes in the tumor microenvironment, such as alterations to immunity and angiogenesis.

### 2.1 Regulation of malignant phenotypes: proliferation, migration, invasion, and apoptosis

Let-7i regulates gene expression to control the processes that underpin malignant phenotypes, such as tumor cell proliferation, migration, invasion, and apoptosis. Let-7i can regulate gene expression indirectly via the classical mRNA regulatory pathway or directly via the non-canonical epigenetic regulation pathway.

#### 2.1.1 mRNA regulation

The regulation of protein levels by specifically binding to the mRNA 3′ UTR is the classic mechanism employed by Let-7i. Let-7i reduces melanoma cell proliferation and metastasis by upregulating KISS1 expression (22), inhibits the proliferation and invasion of osteosarcoma by downregulating the expression of Aurora B (a member of the serine/threonine protein kinase family) (23), inhibits the survival, proliferation, and motility of gastric cancer cells by downregulating the expression of COL1A1 (24), and promotes the DDP-induced apoptosis of esophageal cancer cells by downregulating the expression of ABCC10 (25). In the process of suppressing the occurrence and development of colorectal cancer, Let-7i can not only specifically bind to serine protease (KLK6) mRNA to inhibit its transcription (26) but also inhibits the activity of the ERK signaling pathway by inhibiting the expression of CCND1 (27). In glioblastoma, a study by Xiaopeng Sun et al. found that Let-7i-5p could downregulate the levels of cyclin-dependent kinases (CDK2 and CDK4), cyclin A2, and BCL-2 by silencing GALE, thereby inducing cell cycle arrest and a reduction in proliferation (28). Furthermore, according to experiments by Lobna Elkhadragy, ERK3 and BMI1 are both highly expressed in head and neck cancer and BMI1 upregulates ERK3 by suppressing the expression of Let-7i, ultimately facilitating the migration of head and neck cancer cells (29). Therefore, we speculated that Let-7i can prevent head and neck cancer cells from migrating by reducing the activity of ERK3.

#### 2.1.2 Epigenetic alterations

DNA, histones, non-histone proteins, and a small amount of RNA can all bind and interact with chromatin, which is a linear complex structure containing the genetic material of interphase cells (30). Epigenetics refers to heritable modifications of gene function that ultimately alter phenotype but do not entail changes to the DNA sequence itself. DNA methylation, histone modification, non-coding RNA regulation, and chromatin remodeling are all examples of epigenetic processes. Let-7i may play a tumor-regulating role by modulating epigenetics.

Let-7i acts on histone lysine demethylase to achieve tumor suppression through structural modification. In esophageal cancer, KDM5B, a histone 3 lysine 4 (H3K4) methylation regulator (31), can be downregulated by Let-7i to encourage the tri-methylation of H3K4 (H3K4me3) in the promoter region, consequently promoting the expression of the tumor suppressor SOX17 (32). Overexpressed SOX17 can then silence the tumor promoter GREB1, thereby reducing the proliferation and invasion of esophageal cancer cells and exerting anti-tumor efficacy *in vitro (* 32, 33). In lung cancer, Let-7i enhances DCLK1 expression by interacting with endogenous KDM3A, allowing KDM3A to bind to the promoter region of DCLK1 and removing histone H3K9me2 (34). The enhancement of DCLK1 expression promotes the expression of FXYD3, which reduces the ability of lung cancer cells to proliferate, migrate, and invade, thereby exerting its anti-tumor effect. The above mechanisms have been confirmed *in vitro* and *in vivo (* 34). Yawen Liu et al. suggested that Lin28B upregulates the level of TET3 by blocking Let-7i, while TET3 catalyzes the conversion of 5-methylcytosine to 5-hydroxymethylcytosine, resulting in DNA depletion and pancreatic cell carcinoma (35). Through a feedback mechanism, TET3 and Let-7i can also promote the expression of Lin28B (35, 36).

**Figure 1:**
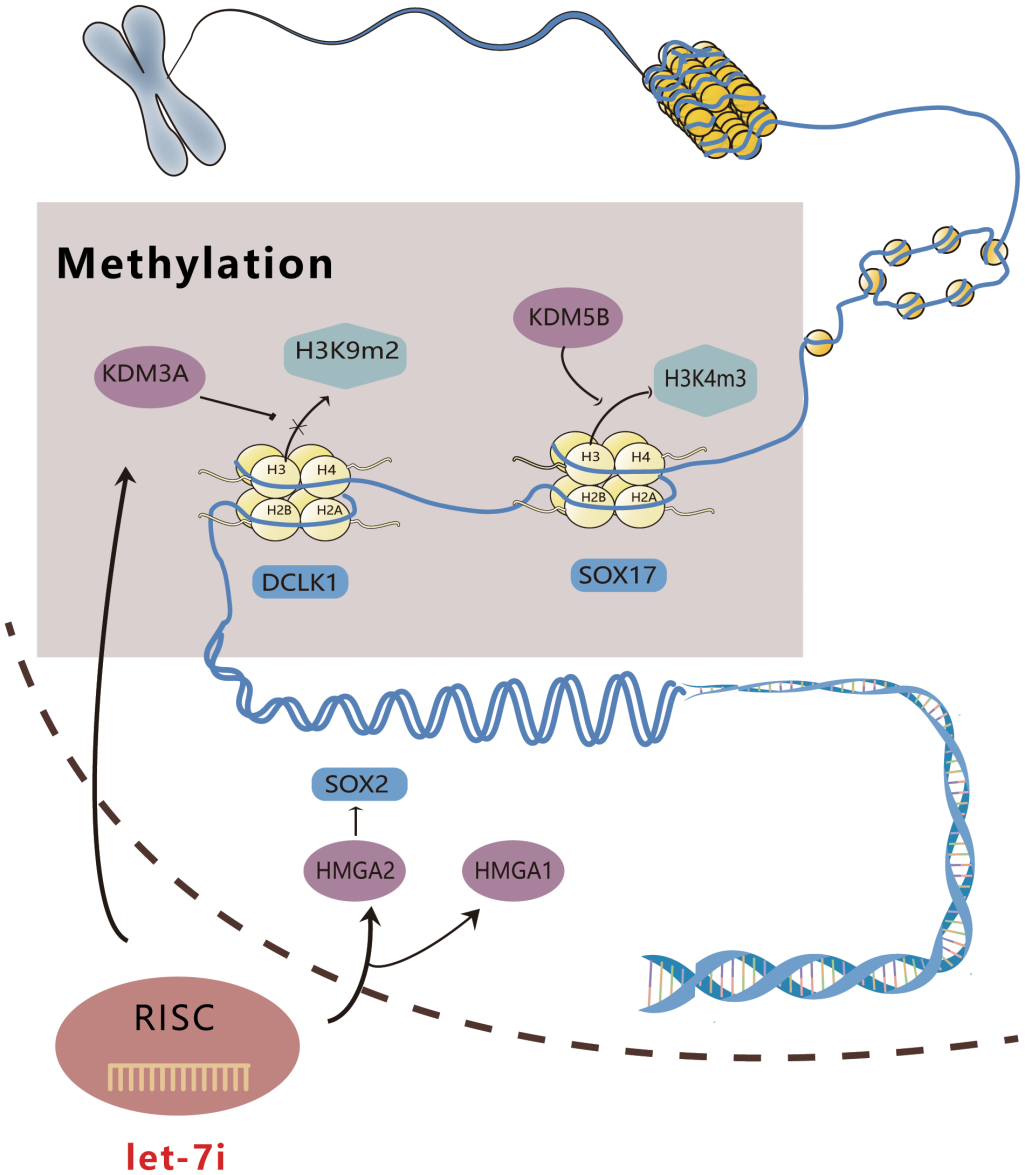
Schematic diagram of Let-7i regulating epigenetics. Let-7i can enter the nucleus to combine with KDM3A and KDM5B, affect DNA methylation, and directly interfere with gene expression. Additionally, it can act on HMGA1 and 2, modify the conformation of DNA, and directly regulate gene expression.

High mobility group proteins A1 and 2 (HMGA1, 2) are members of the HMGA family that are a class of non-histone chromatin structural proteins with no transcriptional activity, which primarily regulate transcription by altering DNA conformation (37). HMGA1 exerts its tumor-regulating effects via multiple pathways, including DNA phosphorylation, acetylation, and methylation (37). Qin et al. illustrated that by targeting HMGA1, Let-7i suppressed the malignant phenotype of bladder cancer cell lines T24 and 5637 (38). According to Ravindresh Chhabra’s study, Let-7i-5p overexpression and SOX2 silencing could both decrease the number of spheroids formed in the cervical cancer cell lines HeLa and CaSki, while HMGA2 and SOX2 expression were significantly reduced in CaSki following Let-7i-5p overexpression (39). HMGA2 has been shown to induce SOX2; therefore, we speculate that Let-7i-5p can disrupt the stem cell phenotype, alter the conformation of DNA, and downregulate SOX2 expression by targeting HMGA2 expression (39–41). **Figure 1** shows a schematic diagram illustrating the way in which Let-7i regulates epigenetics.

### 2.2 Tumor microenvironment pathway regulation

#### 2.2.1 Epithelial-mesenchymal transition and mesenchymal phenotype

Epithelial-mesenchymal transition (EMT) is the process by which epithelial cells lose polarity and transform into motile mesenchymal cells, acquiring a mesenchymal phenotype (42). This process mediates tumor metastasis by blocking connections between cells, reorganizing the cytoskeleton, altering cell shape, and encoding gene expression to enhance cell motility, migration, and invasion. In addition, EMT promotes stem cell likeness and plays a key role in the processes of treatment resistance, embryonic development, and organ fibrosis (42–44). Epithelial cadherin (E-cadherin) degradation is a fundamental mechanistic feature that deconstructs intercellular links and induces EMT, leading to tumor metastasis (42).

**Figure 2:**
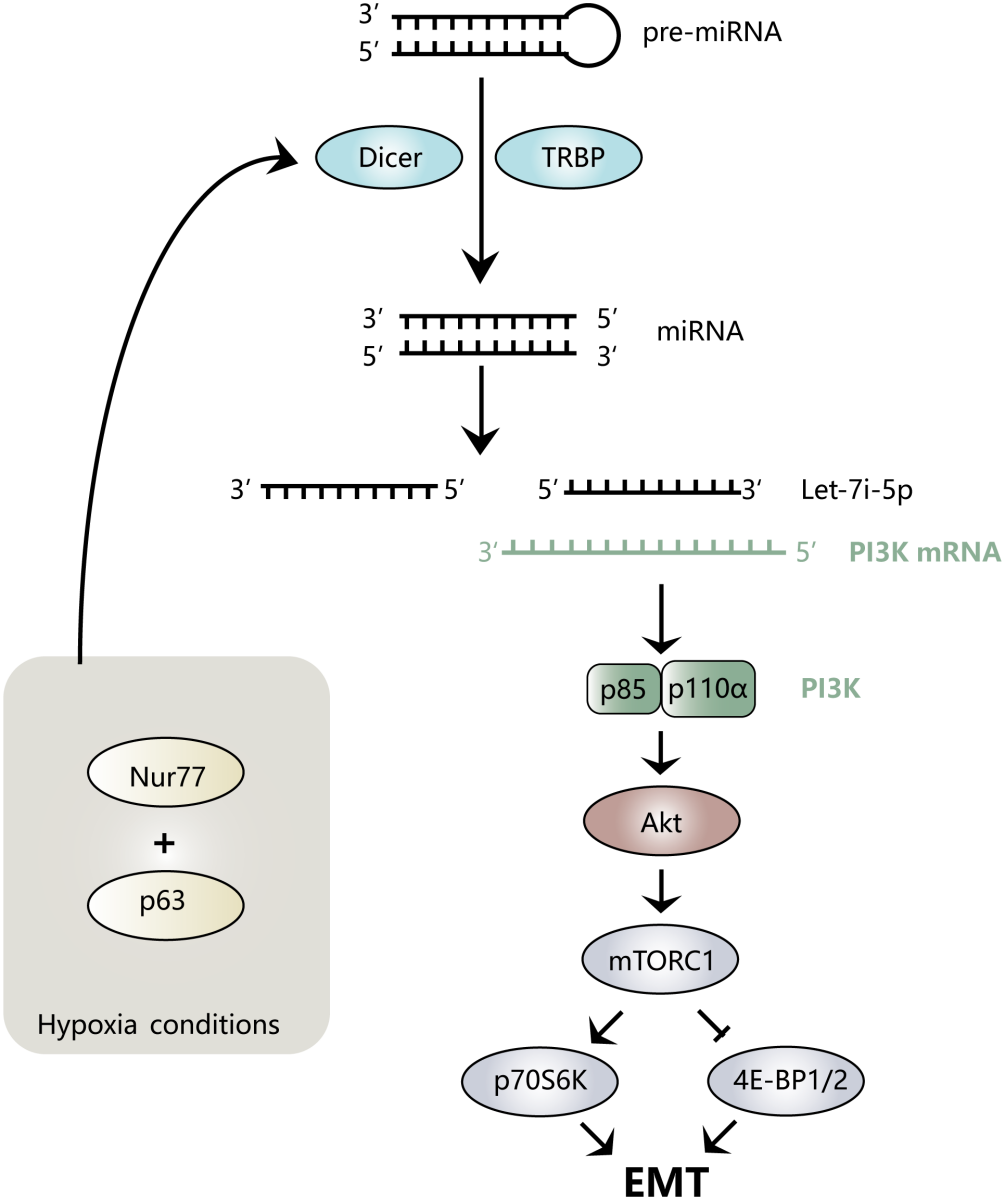
Mechanism diagram of Let-7i regulating EMT. Nur77 binds to p63 under hypoxic conditions, inhibits the maturation of Let-7i-5p, prevents the degradation of PI3K mRNA, activates the Akt signaling pathway, and regulates downstream mTORC1, p70S6K, and 4E-BP1/2, thereby inducing the EMT process.

Hypoxia is a common feature of the tumor microenvironment. It can activate hypoxia-inducible factor-1α (HIF-1α) to further regulate the expression levels of Nur77 (45) and TWIST1 (46). Nur77 is a distinct nuclear receptor, the low expression of which can cause E-cadherin to be downregulated, causing more dispersed colonies to form and triggering EMT and typical mesenchymal morphology (45). Let-7i-5p plays a key role in this process. Under hypoxia, Nur77 interacts with p63 to specifically inhibit Dicer, which affects the maturation of Let-7i-5p from precursor (pre)-Let-7i, resulting in a decrease in Let-7i-5p levels (45). Let-7i-5p binds p110α mRNA on the 3′ untranslated region (UTR) and promotes its degradation, while low expression of Let-7i-5p reduces the degradation of PI3K-p110α to increase its level and activates the Akt signaling pathway. Additionally, the low expression of Let-7i-5p affects the phosphorylation of downstream mTORC1 and its target proteins p70S6K and 4E-BP1, thereby inducing colorectal cancer (CRC) EMT (45). **Figure 2** provides an intuitive illustration of the above mechanism.

TWIST1, a basic helix-loop-helix (bHLH) transcription factor, is a master regulator of EMT (46–48). It is regulated by HIF-1α, regulates BMI1 levels, and cooperates with BMI1 to inhibit E-cadherin expression to induce EMT (49). Let-7i expression can be co-repressed by TWIST1 and BMI1 simultaneously, while low Let-7i levels can increase cell invasiveness. Let-7i downregulation changes the morphology of head and neck squamous cell carcinoma (HNSCC) OECM-1 cells, causing them to adopt an elongated shape with pseudopodia protrusions, which promotes the interstitial cell pattern, ultimately increasing their capacity to move and invade. In addition, downregulating Let-7i increases the expression of NEDD9 and DOCK3, activates RAC1, drives interstitial movement, and further enhances the invasive phenotype (50).

Moreover, in human glioma cells, Yuan et al. confirmed that Let-7i directly targets IKBKE (inhibitor of nuclear factor kappa-B kinase subunit epsilon) to upregulate E-cadherin expression and suppress EMT (51). In endometrial cancer cells, Let-7i is expressed at low levels under the control of DICER1, and low levels of Let-7i have been found to downregulate the expression of EZH2 to affect the methylation of histone H3 at arginine 27 as well as total H3 acetylation, thereby inhibiting the expression of E-cadherin and encouraging EMT (52). In head and neck squamous cell carcinoma (HNSCC), Let-7i inhibits MBP4 to alter cell morphology, turn slender cells round, and decrease interstitial movement, ultimately preventing local invasion (53).

#### 2.2.2 Extracellular vesicles

Extracellular vesicles (EVs), including exosomes and shed microvesicles (sMVs), mediate intercellular trafficking and are crucial for enabling bidirectional communication between cells and the microenvironment at both the paracrine and systemic levels (54). Studies have repeatedly demonstrated the close connection between EVs and the emergence of cancer. EVs transport a wide range of molecules from donor cells to recipient cells, including proteins (such as oncoproteins and oncopeptides), RNAs (such as microRNA and mRNA), DNA fragments, and lipids; this process profoundly alters the phenotype of the tumor microenvironment (54–56). Adeleh Taghi Khani et al. confirmed by *in vivo* and *in vitro* experiments that Let-7i can be delivered by the intercellular delivery system-EV, exerting its tumor suppressive effect in breast cancer cells (57). Experiments conducted by Jiefeng Liu et al. demonstrated that Let-7i inhibits the malignant phenotype of lung cancer through EV transport (34). Additionally, results from a study by Deyi Xiao et al. suggest that Let-7i may act on LIN28B and HMGA2 to alter the expression of EMT markers, thereby inhibiting exosome-mediated melanocyte invasion by suppressing EMT-like effects (58).

## 3 Tumor promoter function

Although Let-7i is widely recognized as a tumor suppressor, increasingly more studies in recent years have found that Let-7i also has a tumor-promoting effect. Moreover, it appears to promote tumor growth and development through different pathways in different tumors.

### 3.1 Classical pathways to modulating malignant phenotypes: proliferation, migration, invasion, and apoptosis

Let-7i promotes hepatocellular carcinoma (HCC) by targeting TSP1. By conducting *in vitro* experiments, Hee Doo Yang et al. found that Let-7i-5p rescued a range of tumor suppressive effects of HDAC6, while the ectopic expression of a Let-7i-5p antisense inhibitor (AS-Let-7i-5p) inhibited tumor cell proliferation, induced apoptosis, and prevented migration under chemotactic stimulation, revealing that Let-7i-5p promotes HCC (20). The thrombospondin-1 gene (THBS1) 3′ UTR was cloned into a reporter vector and detected using an AS-Let-7i-5p dual-luciferase reporter assay, after which, there was an observed increase in the relative luciferase activity (20). In addition, we observed that AS-Let-7i-5p transfection increased thrombospondin-1 protein (TSP1) secretion in the conditioned medium of HCC cells. It has been proposed that Let-7i-5p can interact directly with the transcript 3′ UTR to selectively regulate the expression of THBS1, thereby regulating TSP1 secretion. The inhibition of Let-7i-5p can upregulate the level of TSP1 and inhibit both tumor growth and invasion (20). Therefore, we hypothesized that Let-7i-5p may contribute to tumor growth by suppressing the expression of THBS1 and lowering TSP1 levels. In nasopharyngeal carcinoma (NPC), Let-7i-5p has been demonstrated to act not only as an oncogene to promote cancer but also as a valuable biomarker to evaluate its end stage, predict its recurrence, and predict its metastasis risk. Bo You et al. revealed that Let-7i-5p expression was upregulated in NPC and was significantly associated with clinical stage, recurrence, and metastasis. Patients with a higher ISH staining score exhibited higher Let-7i expression, while patients with higher Let-7i-5p expression displayed worse overall survival (OS) and progression-free survival (DFS) rates (59). Simultaneously, the study confirmed the faciliatory effect of Let-7i-5p on the proliferation and migration of NPC cells through several *in vitro* experiments (59). Results obtained from luciferase gene assays showed that Let-7i-5p binds to the 3′ UTR of ATG10 and ATG16L1, revealing the direct targeting effect on genes (59). Let-7i-5p promotes tumor cell proliferation and migration, while the knockdown of ATG10 and/or ATG16L1 abolished this effect, indicating that Let-7i-5p exerts its effect by controlling ATG10 and ATG16L1 (59). In renal clear cell carcinoma (ccRCC), Let-7i-5p is also highly expressed as an oncogene, and its expression level is strongly associated with the pathological stage. Experiments conducted by Yujie Liu et al. showed that the level of Let-7i differed significantly across different pathological stages and different AJCC stages, allowing it to be used as a prognostic marker for ccRCC (21). Meanwhile, the same research showed that Let-7i-5p can promote malignant phenotypes by directly targeting hyaluronan-binding protein 4 (HABP4) (21). HABP4 is a nuclear and cytoplasmic regulatory protein involved in the regulation of gene expression at the transcriptional and mRNA levels. Additionally, it regulates the cell cycle and apoptosis to modulate cell proliferation (60). Downregulating the level of HABP4 to regulate the cell cycle may therefore be the mechanism by which Let-7i promotes ccRCC (21).

**Figure 3:**
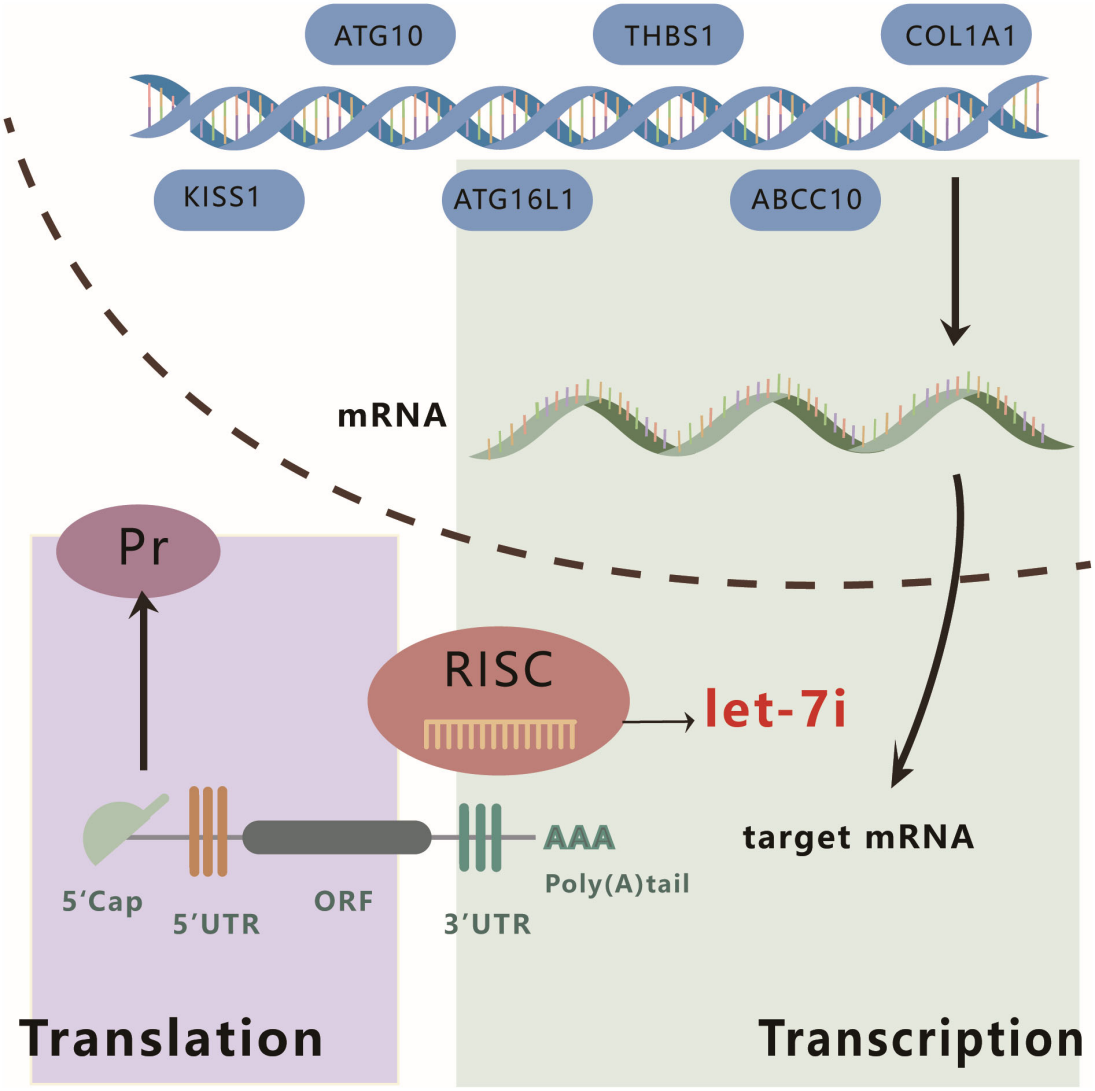
Schematic diagram of the mechanism of action of Let-7i. Let-7i can regulate the expression of KISS, ATG10, ATG16L1, THBS1, ABCC10, and COL1A1 at the post-transcriptional level by binding to the 3′ UTR of mRNA.

Interestingly, Let-7i can promote or suppress hepatocellular carcinoma growth by acting on different targets. Let-7i promotes HCC proliferation and invasion by upregulating the expression of THBS1 and TSP1. Conversely, it also inhibits the malignant phenotype of HCC cells via multiple pathways. A study by Injie Omar Fawzy et al. indicated that Let-7i can inhibit the viability and colony-forming ability of HCC cells either by directly targeting IGF1R or by indirectly reducing IGF1R expression via regulating the expression of insulin-like growth factor 2-mRNA-binding proteins (IGF2BP) 1, 2, and 3 (61). Alternatively, Let-7i can mediate the downregulation of the apoptosis protein Bcl-xL, thereby inhibiting HCC (62). **Figure 3** features a schematic diagram that summarizes the classical mechanism of action of Let-7i.

### 3.2 Angiogenesis and extracellular vesicles

Angiogenesis in tumor tissue is an important prerequisite for rapid tumor proliferation. Tumor tissue blood vessels originate from the pre-existing vasculature and serve as a source of nutrients and oxygen for the tumor cells to ensure their rapid proliferation. The development of vascular architecture in the tumor microenvironment depends on the coordination of pro- and anti-angiogenic factors (63). Hee Doo Yang et al. treated HCC cells with AS-Let-7i-5p and rTSP1, finding that the *in vitro* development of microtubule cells was noticeably suppressed. This effect was successfully rescued by combining the treatment with the TSP1 antibody C-terminal domain to the CD47 receptor (3F352). The research elucidated the following mechanism: the downregulation of Let-7i-5p levels mediates TSP1 binding to the cell surface receptor CD47 to exert anti-angiogenic activity (20). We thus concluded that Let-7i contributes to the promotion of angiogenesis during the development of tumors.

In a study by Hee Doo Yang et al. (20), exosomes were isolated and purified from HCC cell culture medium, from which Let-7i-5p was detected by qPCR, and donor cell fractions were analyzed. The results of the study showed that Let-7i-5p was mainly present in the exosomes but not in the donor cells of HCC cells. The exosomes were then fluorescently labeled with PKH67 dye and incubated with the receptor system. Measurements found that the expression of Let-7i-5p in the receptor cells was significantly enhanced, suggesting that in HCC, Let-7i-5p facilitates communication between liver cancer cells and normal cells via exosomes, subsequently promoting the malignant transformation of cells and the development of cancer (20).

### 3.3 Regulation of autophagy

Autophagy is an intracellular degradation process that fuses autophagosomes and lysosomes by the action of various autophagy genes. It hydrolyzes damaged organelles and macromolecules by hydrolases (64). Autophagy plays a complex dual role in tumors, not only by inducing programmed death to eliminate tumor cells but also by promoting cancer cell-stroma communication to promote tumorigenesis and development, supporting tumor growth in a nutrient-limited environment (65, 66). Cancer autophagy is affected by factors such as nutrient availability, microenvironmental stress, and the immune system (65). Numerous studies have documented how miRNAs regulate autophagy and how autophagy affects tumor progression (67, 68).

In NPC, Bo You et al. found that the transfection of NPC cells with a Let-7i-5p inhibitor could inhibit their proliferation and migration ability via autophagy (59). The research also found that silencing the expression of Let-7i-5p induced LC3 aggregation and increased the number of both yellow fluorescent autophagosomes and red fluorescent autolysosomes in the autophagic flux assay, indicating that Let-7i-5p can inhibit the formation of the autophagy phagosome and inhibit the autophagic flux of NPC cells (59). Furthermore, after knocking out Let-7i-5p, western blot showed that the expression levels of the autophagy marker LC3-II and the autophagy-related gene *ATG5* were significantly increased, while the level of the autophagy substrate p62 was decreased (59). The modulation of autophagy by Let-7i was also observed in non-small cell lung cancer (NSCLC). The transfection of a Let-7i-5p inhibitor into NSCLC cells resulted in an increase in the LC3-II/LC3-I ratio and an increase in the number of autophagosomes, while p62 levels were decreased, suggesting that Let-7i-5p negatively regulates autophagy (69). Taken together, Let-7i-5p exerts a tumorigenic role in NPC through the inhibition of autophagy activity (59).

### 3.4 Regulation of immune escape

As an important part of the immune system, innate immunity is the first line of defense against infection and malignant cell transformation (70). Macrophages can act as antigen-presenting cells (APCs) in innate immunity, processing and cross-presenting antigens to T cells to activate adaptive immunity (71). In addition, macrophages have the ability to mediate phagocytosis, involving multiple cell processes such as target cell recognition, phagocytosis, and lysosomal digestion, which are essential for the programmed clearance of damaged and foreign cells (72). Phagocytosis depends on the relative expression of pro- and anti-phagocytic signals on target cells. Tumor cells have been shown to evade macrophage phagocytosis by expressing anti-phagocytic signals, including CD200 and CD47 (73).

In hepatocellular carcinoma (HCC), TSP1 can prevent the interaction between CD47 and SIRPα, disrupt the “don’t eat me” signal between hepatoma cells and macrophages, and prevent immune escape (20). SIRPα is a signal-regulating protein that is mainly expressed on the surface of myeloid cells such as macrophages. It binds to the transmembrane protein CD47 and is activated to initiate a signal transduction cascade, resulting in the inhibition of phagocytosis (74). It has been reported that cell migration ability was significantly inhibited following the treatment of HCC cells with a Let-7i-5p antisense inhibitor and recombinant TSP1, whereas combined treatment with 3F352 rescued these responses, suggesting the existence of an autocrine/paracrine TSP1-CD47 mechanism in HCC cells (20). Then, co-cultured mouse peritoneal macrophages and HCC cells were treated with a Let-7i-5p antisense inhibitor and recombinant TSP1, and consequently, an increase in the phagocytic index and enhanced macrophage phagocytic activity were observed. This suggests that TSP1 can compete with SIRPα for CD47, convert the CD47-SIRP interaction between HCC and macrophages into the CD47-TSP1 interaction, activate the “eat me” signal, and restart macrophage phagocytosis. However, Let-7i could target and downregulate the levels of TSP1, inhibiting the competitive binding of TSP1, which suppressed the immune response, mediated immune escape, and encouraged the development of tumors (20).

**Figure 4:**
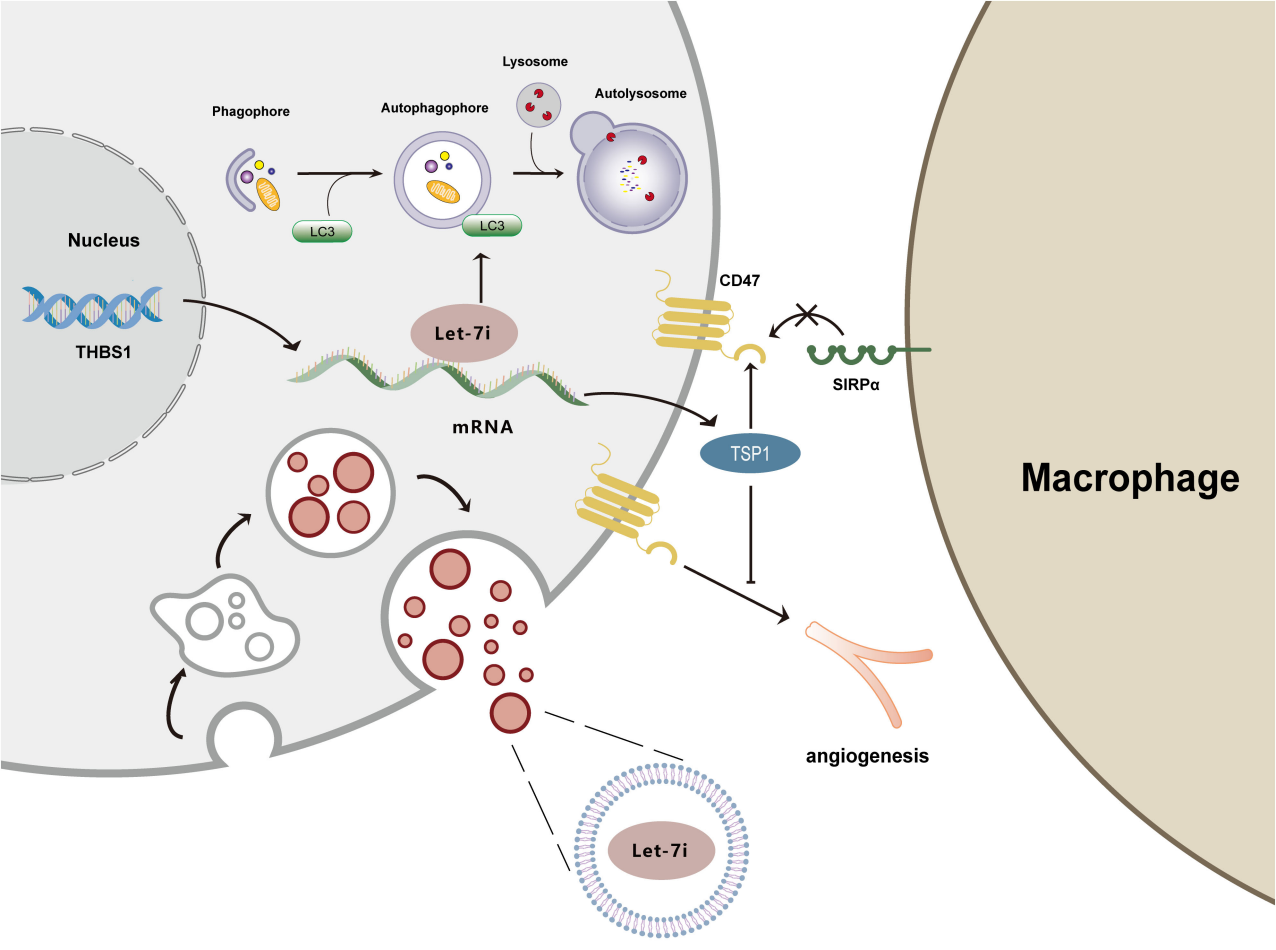
Mechanism diagram of Let-7i regulating autophagy, angiogenesis, immune response, and exosomes. Let-7i suppresses autophagy by reducing LC3 aggregation. Let-7i blocks the immune response and initiates immune evasion by downregulating TSP1. Additionally, Let-7i inhibits the anti-angiogenic effects of TSP1. Furthermore, Let-7i transmits between cells through exosomes.

**Figure 4** illustrates the mechanisms by which Let-7i suppresses immunological response, promotes angiogenesis, inhibits autophagy, and delivers via exosomes.

## 4 Diagnostic and prognostic biomarkers

Although there is an extensive body of research on cancer and a deep understanding of its development, numerous challenges remain regarding its diagnosis. Most cancers occur insidiously but develop rapidly, and when diagnosed, they are often already at an advanced stage, which is greatly related to an untimely diagnosis. Therefore, it is of great significance to improve the diagnostic methods, establish convenient, accurate, and efficient diagnostic biomarkers, detect lesions in a timely manner, and follow up and confirm diagnoses at an early stage.

In almost all cancer types, miRNA signatures are enriched for proteoglycan-related proteins. Proteoglycans are macromolecules that are major components of the extracellular matrix, and alterations in their expression correlate with the prognosis of malignant tumors (75, 76). Specific miRNA signatures regulate proteoglycan and stem cell pluripotency in the tumor microenvironment, which may have profound implications for early cancer detection.

Sathipati et al. (77) suggested that the recognition of Let-7i signatures by CancerSig miRNAs can be used as a basis for predicting the development and stage of various types of cancers, which can help in early cancer identification and stratification. In experiments conducted by Liang Li et al. (78), serum Let-7i was detected in preclinical HCC patients and has the potential to be used in the screening of CHB patients at high risk of developing HCC 6–12 months after the measurement of miRNAs. Cochetti (79) used Let-7i to differentiate patients with prostate cancer from those with benign prostatic hyperplasia and found that the expression level of Let-7i decreased with increasing malignancy of prostate cancer, which led to the suggestion that Let-7i may be a potential marker for high-risk disease. In addition, Let-7i was found to be a potential biomarker for smoking-associated pneumonia (80). The nucleotide diversity of Let-7i can also affect the risk of cervical cancer, head and neck cancer, and many other cancers by influencing Let-7i levels (81, 82).

The issue of tumor prognosis, which considers tumor recurrence and metastasis, is still the focus of attention in the prevention and treatment of malignant tumors, as it seriously affects the survival time and quality of life of affected patients. Therefore, there is an urgent need to identify a biological marker to monitor the effect of tumor treatment and determine prognosis to intervene early, adjust the treatment plan in time, and select the optimal treatment. Let-7i has been extensively studied as a candidate prognostic biomarker for clinical applications. For pancreatic neuroendocrine tumors, Let-7i predicts metabolic aggressiveness and contributes to pancreatic neuroendocrine tumor (PanNET) stratification by peptide receptor radionuclide therapy (PRRT) (83). Let-7i was found to be significantly associated with hepatitis infection and overall survival in patients with hepatocellular carcinoma and was an independent factor in the development of hepatocellular carcinoma (HCC) in patients with chronic hepatitis B (CHB) and chronic hepatitis C (CHC) (84). Let-7i is an early predictor of HCC development after antiviral therapy, and circulating Let-7i levels can be used for the early surveillance of CHB and CHC with HCC risk and as a non-invasive biomarker to predict the risk of hepatocellular carcinoma after antiviral therapy in patients with chronic hepatitis B and C (84). Moreover, Let-7i is associated with poorer overall cancer survival (OS) and could be a potential biomarker for prognostic survival in individuals with tumors (85). Let-7i has been demonstrated to be a good predictor of overall survival (OS) in metastatic renal cancer (86), recurrence-free survival (RFS) in oral cancer (87), progression-free survival (PFS) in advanced ovarian cancer (88), and liver metastasis-free survival (HFS) in colorectal cancer (89). Let-7i not only predicts OS in gastric cancer but also predicts the sensitivity of gastric cancer to chemotherapy (90). Similarly, Let-7i can be predictive of chemotherapy resistance in ovarian and breast cancer cells (88). The potential biomarker role of Let-7i renders it relevant for clinical studies. A proportional risk model for COX has been developed using the expression of miRNAs, including Let-7i, to robustly predict the high and low risk of distant metastasis in nasopharyngeal carcinoma patients (91).

## 5 Clinical target

Owing to the important role of Let-7i in tumorigenesis and development, research on its utility as a therapeutic target is progressively expanding. Let-7i can enable the cell-cell delivery of Let-7i via exosomes (20). Additionally, it can effectively induce dendritic cell (DC) maturation, which plays a key role in generating an anti-tumor immune response. Based on this, Let-7i-modified exosomes have emerged as a primary therapeutic direction, and this technology can be administered by either intramuscular or intraperitoneal injection to target DCs and promote their maturation as well as enhance the proliferation of T-cells and regulate the release of cytokines, thus exerting a powerful anti-tumor response through enhancing the immune response and remodeling the tumor microenvironment (57, 92). Let-7i-modified exosomes also hold promise in the development of a novel cell-free vaccine for cancer therapy (92).

In addition, Let-7i has been extensively studied for its ability to enhance the sensitivity of cancer cells to chemotherapeutic drugs (93). Let-7i can inhibit the transcription of lncRNA XIST and downregulate the expression of XIST (94). LncRNA XIST has been shown to confer chemoresistance to cancer cells via a variety of pathways, including improved DNA repair and apoptosis regulation (93). Therefore, the downregulation of lncRNA XIST can reduce the proliferation and anti-apoptotic ability of lung adenocarcinoma (LAD) cells, enhance LAD sensitivity to cisplatin, and improve the drug resistance of cancer cells (94). Yan-Ling Ren et al. (95) confirmed that propofol is not only useful as an intravenous anesthetic but also exerts non-anesthetic effects by interacting with various signaling pathways, thereby participating in the regulation of various human malignant tumors. Propofol can reduce HOXA11-AS expression and upregulate Let-7i to regulate the expression of ABCC10 and alleviate the resistance of colon cancer to chemotherapy (95). Nenghui Liu et al. illustrated that a MUC1 aptamer-Let-7i chimera can enhance the sensitivity of epithelial ovarian cancer cells to paclitaxel by downregulating the expression levels of cyclin D1, cyclin D2, Dicer 1, and PGRMC1 (96).

## 6 Article summary

By reviewing the role and mechanism of Let-7i in various tumors, we conclude that Let-7i not only plays a tumor suppressor role but also acts as an oncogenic factor to promote the occurrence and development of cancer. Let-7i employs multiple mechanisms of action across different cancers in a cancer-specific manner. Furthermore, for the same cancer cell, depending on the target, it also plays a different role in promotion and inhibition. Multiple processes underlying the cancer phenotype, including cancer cell growth, migration, invasion, apoptosis, stem cell-likeness, epithelial-mesenchymal transition, EV transmission, angiogenesis, immune evasion, and autophagy, are all regulated by Let-7i. Furthermore, Let-7i is a potential biomarker for the diagnosis or prognosis of various diseases. Therapeutically, Let-7i can modulate the anti-cancer immune response by modifying exosomes while also contributing to the sensitivity of cancer cells to chemotherapeutic drugs to varying degrees. The mechanism of Let-7i is complex and detailed. **Table 1** summarizes different aspects of the mechanism of Let-7i in this paper, providing a theoretical basis and reference for the future use of Let-7i as a clinical target in the treatment of cancer.

**Table 1:** Summary of the Let-7i mechanism.

Cancer type	The role of Let-7i	Direct target	Expression status	Downstream pathways involved	Involved phenotype
Hepatocellular carcinoma	Promoter	THBS1	Downregulate	TSP1	Proliferation, migration, apoptosis, angiogenesis, immune escape
	Inhibitor	Bcl-xL	Downregulate		Proliferation, apoptosis
	Inhibitor	IGF1R	Downregulate		Proliferation, migration, Invasion
	Inhibitor	IGF2BPs	Downregulate	IGF1R	Proliferation, migration, Invasion
Nasopharyngeal carcinoma	Promoter	ATG10, ATG16L1	Downregulate		Proliferation, migration
Clear cell renal cell carcinoma	Promoter	HABP4	Downregulate		Proliferation, migration, invasion
Melanoma	Inhibitor	KISS1	Upregulate		Proliferation, migration,
Osteosarcoma	Inhibitor	Aurora B	Downregulate		Migrate, Invasion
Stomach cancer	Inhibitor	COL1A1	Downregulate		Proliferation, migration,
Esophageal cancer	Inhibitor	ABCC10	Downregulate		Apoptosis
		KDM5B	Downregulate	SOX17, GREB1	proliferation, migration, Invasion, apoptosis
Colorectal cancer	Inhibitor	KLK6	Downregulate	Caspase signaling pathway	Proliferation, migration, Invasion, apoptosis
		CCND1	Downregulate	ERK signaling pathway	Proliferation, migration, invasion
		p110α	Downregulate	Akt	migrate, Invasion
Glioblastoma	Inhibitor	GALE	Downregulate	CDK2, CDK4, BCL-2, Cyclin A2	Proliferation, migration, angiogenesis
		IKBKE	Downregulate		migrate, Invasion
Head and neck cancer	Inhibitor	ERK3	Downregulate		Migrate
		NEDD9, DOCK3	Downregulate	RAC1	Invasion
		MBP4	Downregulate		Migrate
Lung cancer	Inhibitor	KDM3A	Downregulate	DCLK1, FXYD3	Proliferation, migration, Invasion
Lung adenocarcinoma	Inhibitor	XIST	Downregulate		Proliferation, apoptosis, drug resistance
Pancreatic cancer	Inhibitor	TET3	Downregulate		Proliferation, invasion
Bladder Cancer	Inhibitor	HMGA1	Downregulate		Proliferation, migration, Invasion
Cervical cancer	Inhibitor	HMGA2	Downregulate	SOX2	Cell stemness
Endometrial cancer	Inhibitor	EZH2	Downregulate		Invasion

## Author contributions

The idea comes from ZC and JZ, the article is written by JZ, article modification is by HX, all authors reviewed the article. All authors contributed to the article and approved the submitted version.
